# A Multimodal Sensing Platform for Interdisciplinary Research in Agrarian Environments

**DOI:** 10.3390/s22155582

**Published:** 2022-07-26

**Authors:** James Reynolds, Evan Williams, Devon Martin, Caleb Readling, Parvez Ahmmed, Anders Huseth, Alper Bozkurt

**Affiliations:** 1 Department of Electrical and Computer Engineering, North Carolina State University, Raleigh, NC 27695-7911, USA; jlreyno4@ncsu.edu (J.R.); ewillia7@ncsu.edu (E.W.); dmarti22@ncsu.edu (D.M.); cmreadli@ncsu.edu (C.R.); pahmmed@ncsu.edu (P.A.); 2 Department of Entomology and Plant Pathology and North Carolina Plant Sciences Initiative, North Carolina State University, Raleigh, NC 27695-8208, USA; ashuseth@ncsu.edu

**Keywords:** corn earworm, bivalve, testing platform, wireless, Internet of Things, sensor development, agriculture, aquaculture

## Abstract

Agricultural and environmental monitoring programs often require labor-intensive inputs and substantial costs to manually gather data from remote field locations. Recent advances in the Internet of Things enable the construction of wireless sensor systems to automate these remote monitoring efforts. This paper presents the design of a modular system to serve as a research platform for outdoor sensor development and deployment. The advantages of this system include low power consumption (enabling solar charging), the use of commercially available electronic parts for lower-cost and scaled up deployments, and the flexibility to include internal electronics and external sensors, allowing novel applications. In addition to tracking environmental parameters, the modularity of this system brings the capability to measure other non-traditional elements. This capability is demonstrated with two different agri- and aquacultural field applications: tracking moth phenology and monitoring bivalve gaping. Collection of these signals in conjunction with environmental parameters could provide a holistic and context-aware data analysis. Preliminary experiments generated promising results, demonstrating the reliability of the system. Idle power consumption of 27.2 mW and 16.6 mW for the moth- and bivalve-tracking systems, respectively, coupled with 2.5 W solar cells allows for indefinite deployment in remote locations.

## 1. Introduction

The Internet of Things (IoT) community and ecosystem have paved the way for the distributed deployment of sensors and systems in large quantities and across multiple locations. Interest in merging IoT systems into agriculture applications has increased recently [1,2,3,4,5,6,7,8,9,10,11]. While this promises to be a significant data-gathering opportunity for agricultural and environmental researchers, many existing systems come geared towards individual hobby and backyard gardening applications, requiring special attention towards proper calibration for long-term scientific use. Some of these hardware and software implementations are also poorly documented or constructed and difficult to reproduce. The commercially available systems are either too expensive or limited to sense only a certain number of parameters (Table 1).

**Figure 1:**
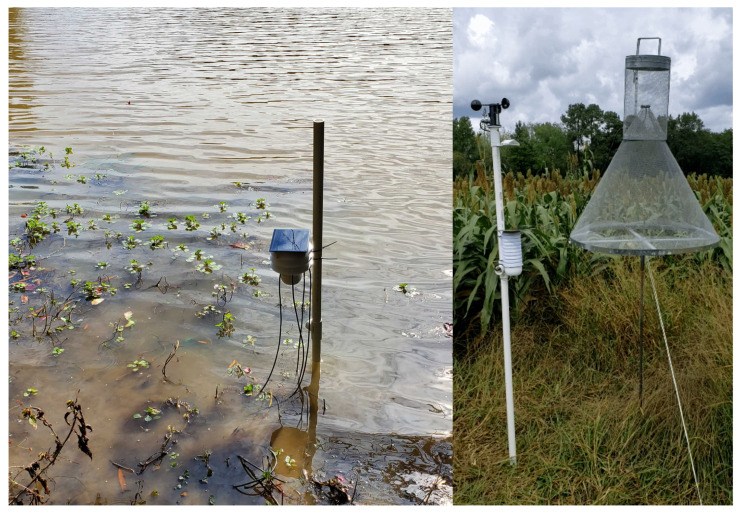
The system as deployed to measure bivalve activity (**left**) and moth activity (**right**).

There is a need for modularity and flexibility in IoT sensor systems to support interdisciplinary research ideas in addition to being low-cost, easy to manufacture, and reliable for scaled up, widespread field deployment. To address these needs, in this work, we present an embedded systems platform and accompanying sensors using standard manufacturing techniques and lower-cost electronic components. To demonstrate its modular flexibility supporting new application-specific needs, we added a set of sensors to track the emergence of corn earworm (*Helicoverpa zea* Boddie) moth populations in agrarian fields and the measurement of bivalve, particularly *Elliptio complanata*, gaping angle in aquatic environments (Figure 1).

IoT devices can provide a better understanding of pupal emergence patterns and adult insect movement, which is critical because these lepidopteran pests impact many crops over most of the growing season [12,13]. The current challenge is improving the tedious and time-consuming process of trapping and counting moths (or other insects), which is further exacerbated by the geographic spread and scale involved with obtaining accurate and comprehensive data. Existing trap networks utilize either species-specific male sex pheromones or blacklights to trap insects. Quantifying moths in these systems is time-consuming and requires specific identification skills. Proposed solutions primarily involve machine- or computer-vision methods [14,15,16,17,18,19,20,21,22]. While they have a range of advantages, the main drawbacks for their field deployment are the increased system cost and high bandwidth requirements in locations where high-speed data are generally unavailable. Optical and acoustic methods also exist [23,24], and these are much better suited for larger-scale deployments.

The study of aquacultured or naturally occurring bivalves is also an active area of research to understand the spread of environmental pollution and improve the production efficiency of aquacultured products. These bivalves play an especially important ecological role in the North American aquatic ecosystem, but many species are currently endangered [25]. The study of their activity and their environment through the use of IoT devices will contribute to their conservation effort [26].

## 2. Materials and Methods

### 2.1. Electronic Layout

The system is designed to be housed in a Stevenson-screen-type enclosure that is either commercially available (925–1418, La Crosse Technology, La Crosse, WI, USA) or can be 3D-printed in a similar shape using acrylic styrene acrylonitrile. An enclosure allowing air to pass through provides more reliable environmental sensing, ensures the components do not overheat, and also limits the effect of solar radiation on the temperature measurements. The enclosure and sensors can be mounted on polyvinyl chloride piping for an inexpensive but reliable stand that can endure UV light exposure over long periods of time. The pipe can be embedded in the soil or riverbed. If the ground is too hard, a metal rod, such as a piece of rebar, can be hammered into place with the piping placed over it. Wires can also be routed through the pipe as needed if a tee socket is used.

**Figure 2:**
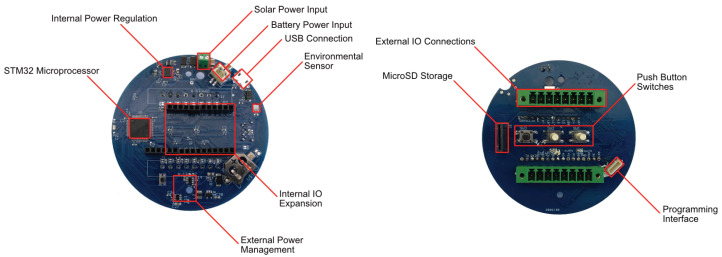
An overview of the motherboard that serves as the central hub of the system.

The presented electronic system is built on a custom printed circuit board (PCB) acting as a motherboard that is mechanically assembled on the base of the Stevenson-type enclosure. This motherboard includes a microcontroller, power circuitry, battery and solar terminals, a backup battery for the real-time clock (RTC), environmental (temperature, barometric pressure, and humidity) sensors, and a secure digital (SD) memory card slot (Figure 2). It also has several terminal blocks for internal expansion of the board within the housing and external expansion by connecting sensors placed outside the housing. The external connections use two eight-pin pluggable screw terminals. These, along with the SD card slot and buttons, are protected from environmental damage by a flange on the base. The motherboard is coated with a layer of synthetic rubber and acrylic to prevent high humidity and condensation from damaging it.

**Figure 3:**
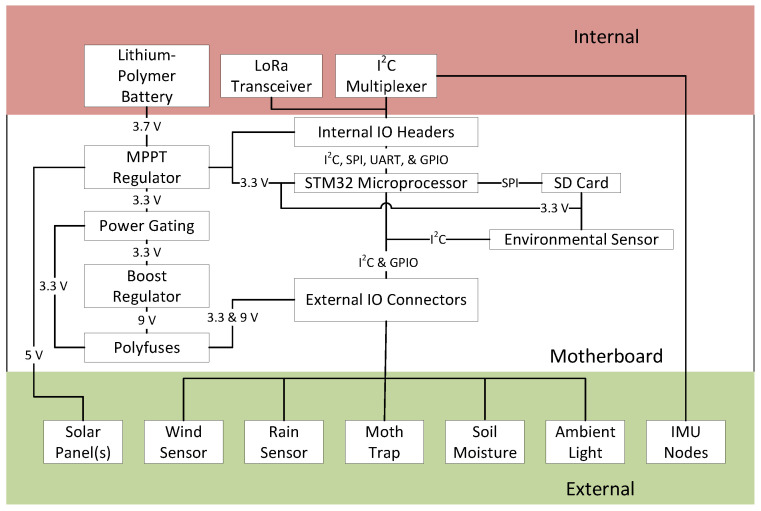
A block diagram of the main system components and their connections.

For internal connections to allow the addition of more sensors and wireless transceivers, we used 2.54 mm female headers providing input/output (IO) connections for several different communication buses and optional analog measurements via the built-in 12-bit analog-to-digital converters (ADC). These headers also match the footprint of commonly used and commercially available embedded systems to build IoT sensor systems, thereby providing a standardized cross-compatibility (e.g., AirLift FeatherWing, Adafruit Industries, New York, NY, USA). This also provides generational and developmental consistency, where sensors remain compatible over time. For example, we demonstrated an earlier system focused on the monitoring of plants [27] that consumed twice as much power as this presented system. Sensors from it, such as the one for bioimpedance measurements, are compatible with this system to allow for even more experimental capabilities in the future. The overall layout of the system with some of its potential expansion options is shown in Figure 3.

### 2.2. Processing

We used STM32L series microprocessors at the core of the system. Its important features include support for an RTC, sufficient IO to support the system, and reduced power consumption—10× lower during sleep than some microprocessors (e.g., SAM D21/DA1 family, Microchip Technologies Inc, Chandler, AZ, USA). Significant advantages include the way the embedded software is used and support for a real-time operating system. While Arduino-based development allows for easy and rapid prototyping, it is less efficient both in terms of computation as well as power consumption. It also lacks the variety of application options available for the STM32L series. We used the LQFP64 footprint as there are many different pin-compatible chips in the STM32L series that can function in this system, which is advantageous during a supply-chain shortage.

Data can be transmitted wirelessly using an expansion module and/or stored on an SD card. The system uses approximately 64 bytes to record a complete set of measurements during each sampling event. With a 1-minute interval between environmental measurements, the system can theoretically fit over 40 years worth of weather data on just a 1 GB SD card.

This design was tested with a long range radio (LoRa) transmitter (SX1276, Semtech Co., Camarillo, CA, USA) mounted via the internal expansion and using a simple quarter-wave ground plane wire antenna, following the recommendation of the device datasheet and in order to reduce costs while still achieving acceptable performance levels. We received data from these systems at distances over 4 km away without any line of sight. This system is also capable of supporting cellular connectivity such as LTE.

### 2.3. Sensors

The system design supports a multitude of sensors, including those we previously documented [27]. Soldered directly into the motherboard system is an environmental sensor (BME680, Bosch Sensortec, Reutlingen, Germany) that records ambient temperature, humidity, barometric air pressure, and volatile organic compounds. The sensing range of this commercially available sensor can be found under the system specifications table (Table 2). The accuracy numbers for each sensor are reported directly from the parts’ datasheets available online from the manufacturer. These datasheets also provide detailed information about the definition of the accuracies and how it is measured. We also assembled three external sensors for wind, rain, and soil sensing. An anemometer (1733, Adafruit Industries, New York, NY, USA) interfaces with the main unit to measure wind speeds up to 70 m/s with an accuracy of 1 m/s and a resolution of 0.1 m/s. In order to conserve power and because the anemometer generates a DC output, the ADC can sample the electrical signal slowly. To detect the presence of rain, a parallel LC resonator was designed where the capacitive component was printed on a PCB as interdigitated fingers. The concept behind this approach is that the transmission of a high-frequency pulse width modulated (PWM) signal on one end of the resonator will cause the output on the other end of the resonator to decrease proportionally to the level of wetness of the sensor. The sensor station connects both the PWM inputs and output connections of the LC resonator to the main unit.

We designed the system to be compatible with three separate soil moisture probes. The commercial sensor (ECH_2_0 EC-5, METER Environment, Pullman, WA, USA) has two electrode prongs and connects to the microcontroller’s ADC. The second probe is an open-source design (I2C soil moisture sensor, Catnip Electronics, Vilnius, Lithuania) with two metal traces on a single circuit board resulting in a slightly different measurement profile compared to the EC-5. It utilizes a 16 MHz square wave generated by the onboard microcontroller in combination with the built-in ADC. The third probe is our custom design printed on a PCB that has an outline similar to that of the EC-5. A 74.25 MHz square wave generated by an oscillator (DSC1033, Microchip Technology, Chandler, AZ, USA) is filtered by a 510 Ω resistor in series with the two conductive legs of the probe acting as a capacitor. The output of this filter (between the resistor and the grounded capacitor) is connected to a differential ADC (LTC2453, Analog Devices, Wilmington, MA, USA). Diodes and capacitors on the ADC input terminals create a DC voltage equivalent to the amplitude of the AC signal. The signal amplitude is inversely proportional and decreases with an increasing dielectric from additional soil moisture. The accuracy and resolution of these sensors for measuring volumetric water content are heavily dependent on conditions and the calibration used. The probe also has a temperature sensor (ADT7410, Analog Devices, Wilmington, MA, USA) to measure the soil surface temperature with an accuracy of ± 0.5 ${}^{\circ }C$ and a range from −55 ${}^{\circ }C$ to over 60 ${}^{\circ }C$.

**Figure 4:**
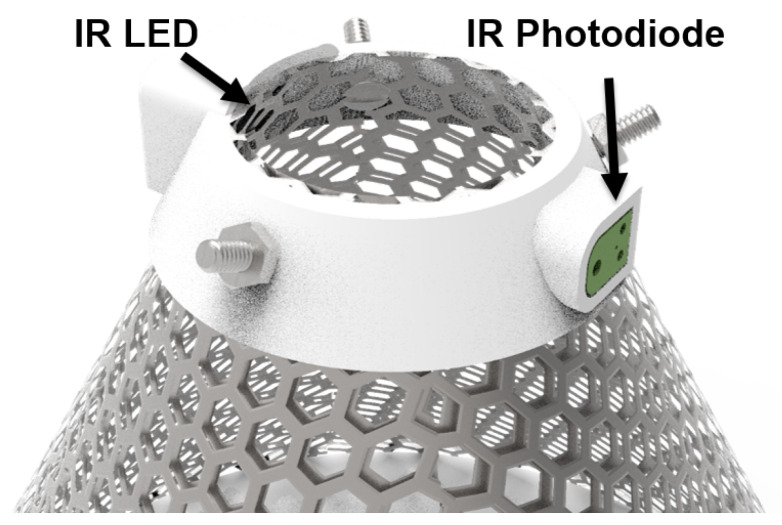
A render of the light emitting diode and photodiode arrangement for counting corn earworm moths as they enter the trap via the funnel.

To demonstrate the versatility of the system to support new data acquisition solutions, we designed a moth-counting sensor for standard moth traps and connected it to the system. An infrared (IR) beam sensor consisting of an IR light emitting diode (LED, APT1608F3C, Kingbright, Taipei, Taiwan) and IR receiver (TSOP36238TR, Vishay Semiconductor, Malvern, PA, USA) pair is employed to count the number of moths entering a pheromone trap [28]. To reduce power consumption, the IR LED is modulated at 38 kHz with a 10% duty cycle. This design consideration lowers the power consumption of the sensor while also reducing the confounding factor of the slowly changing ambient sunlight to improve the signal-to-noise ratio (SNR) of the beam sensor. The beam sensor is mounted on the cone of the moth trap, as shown in Figure 4. A weatherproof cable allows the communication of signals and the transfer of power from the main unit.

As a second application, we connected a set of external inertial measurement units (IMUs) attached to bivalves to measure their gaping activity. Two triaxial accelerometers (LSM303, STMicroelectronics, Geneva, Switzerland) track the bivalve shell movement and form a sensor node that calculates the opening angle between the two shells. Four sensor nodes (8 IMUs) are connected in parallel to an I^2^C multiplexer (PCA9547, NXP Semiconductors N.V., Eindhoven, Netherlands). The four nodes are wired to the I^2^C bus using stranded CAT6 cabling that is soldered to the accelerometer modules and held in place on the multiplexer board by screw terminals. The twisted pairs improve the reliability and integrity of the connections over previous straight cabling. Each accelerometer module is waterproofed using a coating of synthetic rubber and acrylic and hardened using a thermoplastic shell. The I^2^C multiplexer is mounted internally via the stacking headers. As such, the system has the capability to support several multiplexer boards with the potential to obtain measurements from up to 32 bivalves.

**Figure 5:**
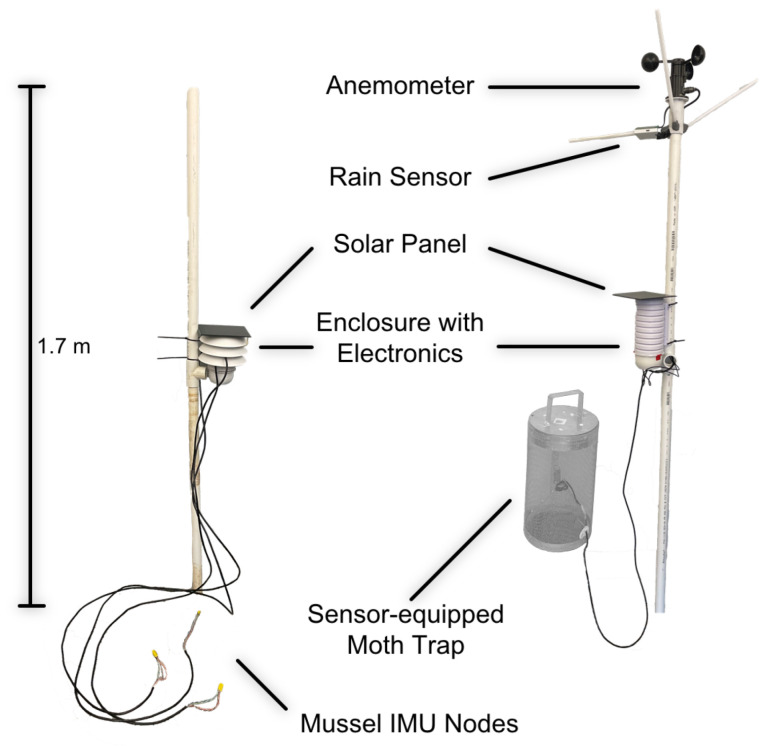
A diagram of the system in its two presented configurations.

Through careful component selection (Table 3) and a custom design process, the cost per system is less than USD 150 per system in its full configuration (Figure 5) for bivalve measurements (which uses just the environmental sensor, accelerometers, multiplexer, and LoRa transceiver) or moth detection (which includes just the environmental sensor, moth trap hardware, rain sensor, and anemometer).

### 2.4. Power

Depending on the available sunlight and the application needs, the system is powered by energy from one or two (connected in parallel) 2.5 W solar panels during the day. The solar panels also charge a battery to power the system at night. A single integrated circuit (LTC3106IUDC, Wilmington, MA, USA) handles the optimization of the solar input, charging the battery, regulating the 3.3 V output power rail, and switching between power inputs. Using a 1600 mAh battery, the system can function indefinitely until a parts failure occurs. A 9 V boost regulator (MIC2288, Microchip Technology, Chandler, AZ, USA) provides power for sensors that require the higher voltage, such as the anemometer. Power to these sensor devices is controlled in two ways. First, MOSFET switches (TPS22918, Texas Instruments, Dallas, TX, USA) allow the microcontroller to selectively disable power to subcircuits on the power rails through power gating. This improves energy efficiency when accessories enabled by these subcircuits are not in use. Second, the output power connections are protected with polyfuses (PRG15BC4R7MM1RC, Murata Manufacturing Co., Kyoto, Japan), which prevent not only the internal regulators from being overloaded but also fire hazards in the environment. They work by transitioning to a high-resistance state if too much current is drawn by the subcircuit. However, the polyfuse will reset when the current load is reduced to acceptable levels. The system supports single-cell lithium-based batteries in either a cylindrical (18650: 18 mm× 65 mm) or flat cell (with a micro JST connector) form factor.

The two configurations have different power consumption characteristics that correspond to their unique modes of operation and attached sensors. The moth system is either measuring environmental data, recording a moth trap trigger, resetting after a trigger, or in a reduced power (sleep) mode. The moth configuration uses the anemometer and rain sensor, and these require longer sampling times. Enabling power to the anemometer results in a voltage and current spike. The additional complexity due to this behavior is offset by the power savings from power gating, since the current draw from the anemometer is quite significant. When the moth trap is triggered, it caches the time and writes to the SD card when the cache is full. A memory size of 1 GB would be sufficient to store 40 years of continuous data with the current resolution. The peak current draw during this mode is caused by writing the data to the SD card. While this occurs, and then for 250 ms after the data is saved, the system ignores any inputs from the trap to prevent the same moth from triggering the trap multiple times when it is on the edge of the detection area. This technique results in a timeout period with slightly higher power consumption than the baseline sleep state. This baseline sleep energy usage is higher than that of the bivalve configuration because the moth trap is always active. Its infrared LED PWM frequency is higher than the maximum frequency of the low-power clock. This prevents the microprocessor from utilizing its more efficient low-power states, which can disable most of the circuitry on the microprocessor but leave just the RTC and interrupt controller active. In both configurations, a timer based on the RTC triggers an interruption to wake up the system at regular intervals to take environmental measurements.

The bivalve configuration, on the other hand, has three other distinct modes: measuring and transmitting environmental data, obtaining accelerometer data, and wirelessly transmitting the bivalve angle data. While each of these can be optimized such that the power is minimized for the required wireless transmission rate and distance as well as accelerometer accuracy, the transceiver was set to use its highest transmit power, and the accelerometers were set to their highest accuracy mode so that the measured power consumption was close to its maximum expected for field deployment.

### 2.5. System Testing

To ensure the system can endure relatively harsher field conditions, we performed two sets of experiments during the final stage of system development. The first test involved placing two systems (just the motherboard with the environmental sensor, a LoRa transmitter module, a battery, an enclosure, and no external sensors) in a closed glass and acrylic chamber ( 77 cm× 32 cm× 32 cm) and heating the chamber from 32 ${}^{\circ }C$ to over 50 ${}^{\circ }C$ for 2 h using a ceramic heater (11-300-49SHP, Fisher Scientific, Pittsburgh, PA, USA) and a 10 cm fan. It took around 45 min for the heater to ramp up to 50 ${}^{\circ }C$ at the beginning of the test. For the second test (for humidity), a third and identical system was included due to availability and an expected more non-uniform distribution of the humidity. These three individual and identical devices had the same configuration and location as the first test. This second test involved saturating the chamber air with moisture using a humidifier (HG-JSQ01W, Zhajiang Huaguang Electric Appliance Group, Cixi, China), 10 to 40 cm away from the devices, such that condensation formed on all the surfaces. After saturation, the chamber was left stationary for 1 h. Then, the air was saturated again, but a 10 cm fan in the chamber maintained continual air circulation so that condensation was less likely. Using the LoRa modules, the systems transmitted their environmental measurements along with an identifier that would indicate if the system had reset.

As part of the next stage of testing, two systems configured for moth detection were placed in a greenhouse for 12 days to test their durability in a relatively controlled environment. One solar panel was placed in a shaded area to simulate reduced ambient light. These devices were splashed during the plant watering, to evaluate IPx4 compliance. During this testing, the devices were able to save the environmental data internally and record environmental information every minute. To test the bivalve accelerometers’ long-term drift, the four nodes were attached together, mimicking the layout on a bivalve, and placed on an experimental benchtop for approximately 5 days. The data were sampled and transmitted wirelessly every 10 min using LoRa on the industrial, scientific, and medical (ISM) band at 915 MHz in the United States. While the frequency band is different in other continents or countries, it is relatively easy to swap the LoRa board with the frequency-compatible ones. The processing of the received signals involved calculating the angle between the accelerometers in each node and then normalizing it to 0 degrees by subtracting the mean of the entire dataset of each node.

The system power measurements were performed using a source meter (2450, Tektronix, Beaverton, OR, USA) set at 5 V and connected to the solar power input without a backup battery. The source meter sampled the current draw at 14.5 Hz. The accelerometers had a sampling rate of 100 Hz during these measurements. For the bivalve system, the LoRa module had a transmit power of + 20 dBm and a spreading factor of 7. The bivalve configuration power estimate was based on measurements every 10 min. The moth system was configured to save its data on an SD card, which occurred every time the device collected sensor data. The hourly power consumption of the moth configuration was calculated with an environmental sensor measurement every 10 min and moth trap was manually triggered (simulating moth entry) 10 times every hour. The solar panels were tested by using the system as a load in three different settings: twilight, cloudy, and full sun. A multimeter (U1232A, Tektronix, Beaverton, OR, USA) measured the current to the system from the panels as well as the voltage across the panel terminals. A light meter (HS1010, Sunche, Shanghai, China) recorded the illuminance on the panels.

We would like to note that we did not need to characterize individual sensor operation as we primarily used commercially available sensors. Our measurements were focused on the verification of the embedded systems integrated with multiple sensors.

## 3. Results and Discussion

**Figure 6:**
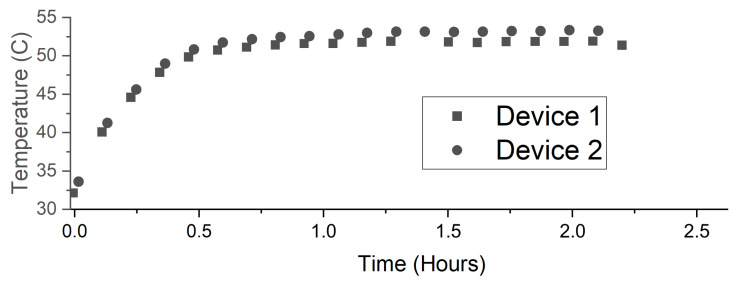
The example temperature measurements from two identical sensing systems during the high-ambient-temperature test where the ambient temperature was ramped up from approximately 32${}^{\circ }C$ to 50${}^{\circ }C$ over a duration of 45 min in a closed chamber.

**Figure 7:**
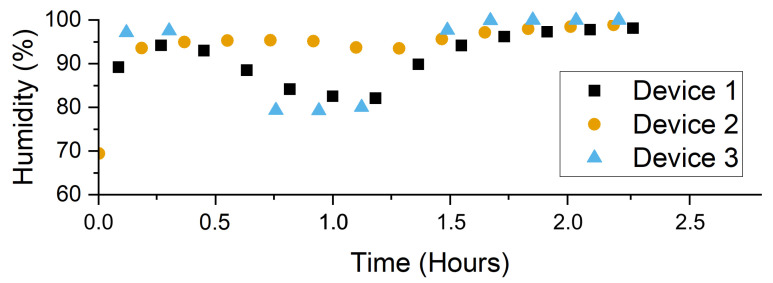
The humidity fluctuations in the chamber as measured by three identical devices during the humidity test. Device 2 was physically closer to the humidifier, thus reflecting higher humidity levels.

The devices achieved a successful operation during the temperature and humidity tests (Figure 6). The humidity test helped us to identify the water insulation weaknesses and to reinforce these parts with additional insulation. Even when the condensation eventually found a weakness in the insulation of one of the systems and shorted electrical contacts, the embedded software reset the system, which recovered in less than one hour to continue transmitting for the remainder of the experiment. The system is sensitive enough to detect the higher humidity closer to the humidifier when the fan was not active, as demonstrated by the second device (Figure 7).

**Figure 8:**
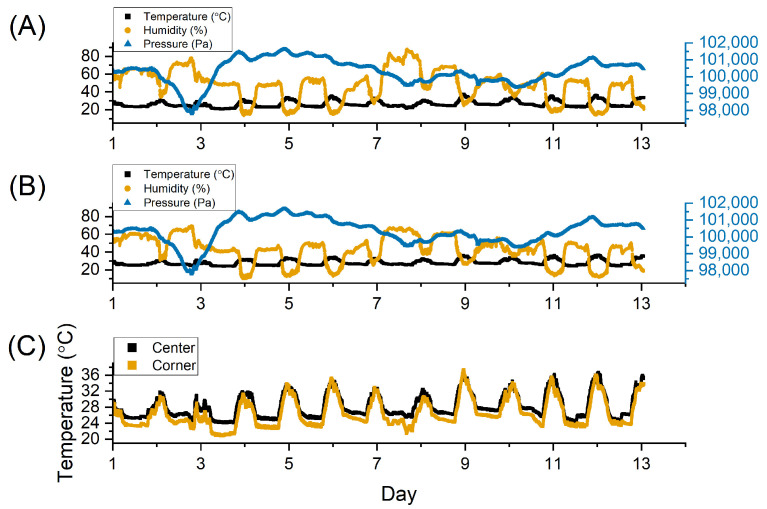
The environmental conditions as measured in the corner of a greenhouse (**A**) and the center of a greenhouse (**B**) with the temperatures of the two locations compared (**C**).

The greenhouse data match with the expected trajectories (Figure 8). The device in the center of the greenhouse measured an average temperature of 28.2 ± 3.02 ${}^{\circ }C$ and an average relative humidity of 42.8 ± 15.0%. In contrast, the edge of the greenhouse had an average temperature of 26.5 ± 3.52 ${}^{\circ }C$ with an average relative humidity of 47.9 ± 17.1%. The average barometric air pressure for each was 100,336.5 ± 744.7 Pa and 100,322.2 ± 738.4 Pa, respectively. The center of a greenhouse is better controlled and isolated, so there would be less variation in the temperature. The air pressure, however, matches closely because the unpressurized room is expected to mirror atmospheric pressure. The devices functioned continually without any resets or errors across the 12-day testing period.

**Figure 9:**
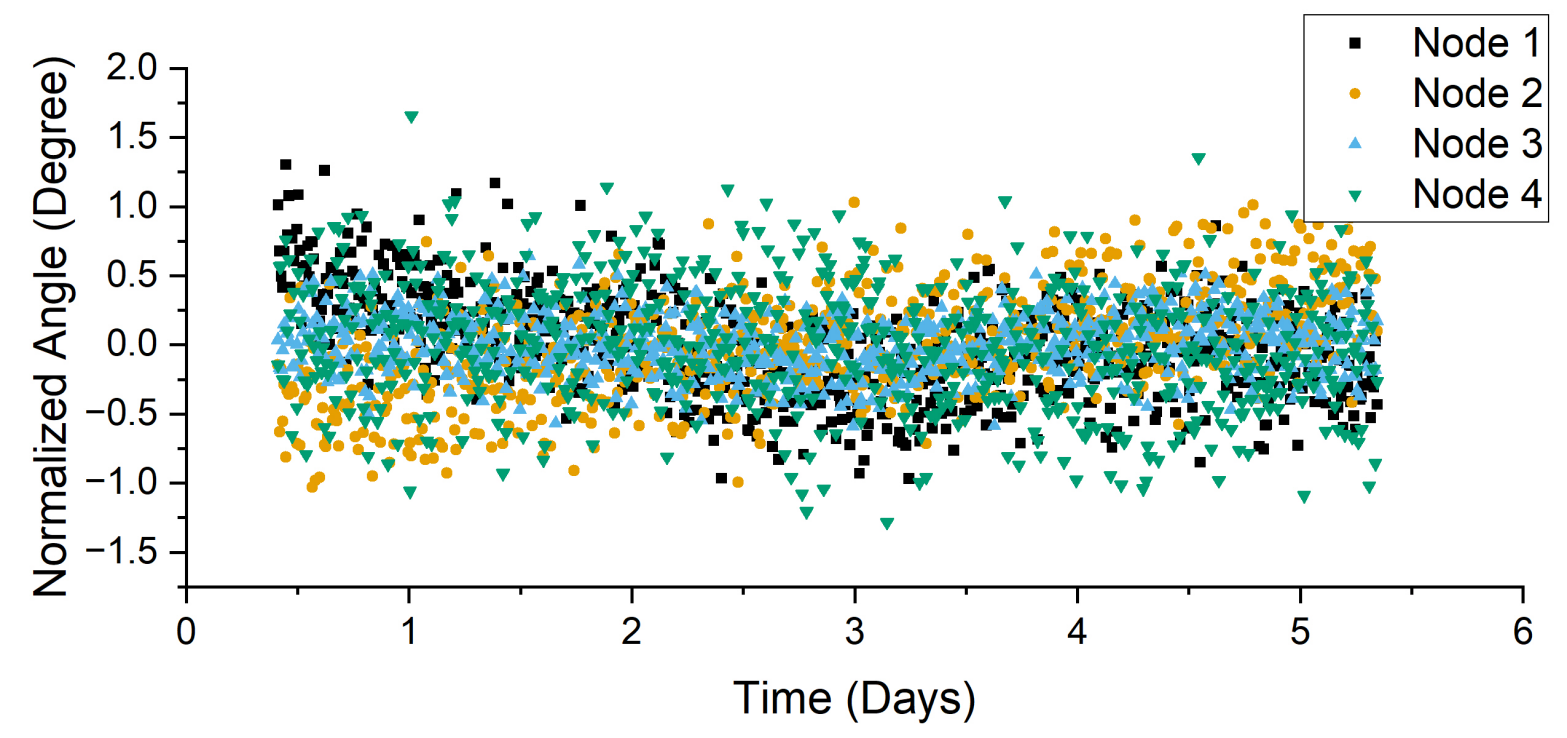
Normalized angle data over 5 days for 4 stationary nodes, demonstrating their stability and noise characteristics in a deployment configuration.

The bivalve measurement configuration demonstrated long-term stability (Figure 9). The standard deviation across the four pairs was 0.74 ${}^{\circ }$. The average range was 2.15 ± 0.61 ${}^{\circ }$. The average interquartile range was just 0.49 ${}^{\circ }$. The average coefficient of skewness was 0.098. These metrics can potentially be improved through increased sampling and averaging as needed for the specific behavior being observed. However, given that bivalve movement is generally greater than this range (around 10 ${}^{\circ }$), the noise levels are acceptable [29].

**Figure 10:**
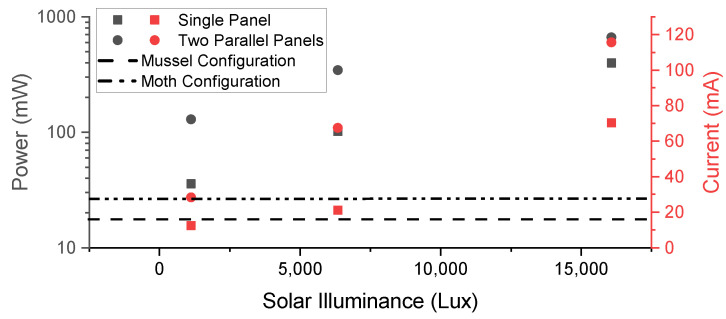
A comparison of the power and current provided by one or two solar panels at different light exposure levels with the idle power consumption of the system in each configuration.

**Figure 11:**
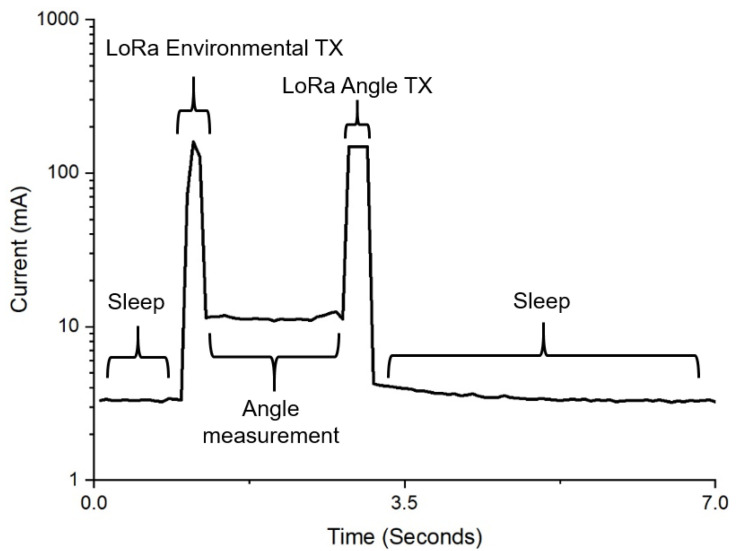
A graph of the current consumption of the system when configured for measuring bivalve activity.

**Figure 12:**
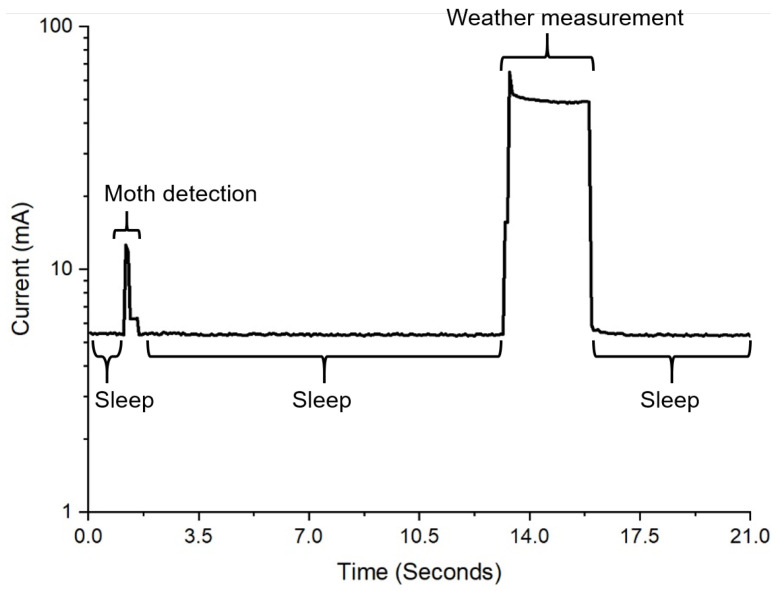
A graph of the amount of current used by the system in its moth detection configuration.

The moth configuration had a higher idle power consumption of 27.2 mW in contrast with the bivalve configuration at just 16.6 mW. Both, however, are sufficiently low enough to enable the long-term solar-powered deployment of these systems (Figure 10). Table 4 and Table 5 present a detailed analysis of the current draw and energy consumption of each key state within the two system configurations, as illustrated by Figure 11 and Figure 12. The total energy required was 27.7 mWh for the moth configuration and 17.2 mWh for the bivalve configuration. The power usage of the system during LoRa transmission depends on the data size. In the case of the bivalve system, this is evident in the difference in current peak widths between the environmental data transmission and the accelerometer data transmission. As the amount of data increases, this sustained power consumption will increase. The average power for each system could be reduced through more aggressive caching, which would reduce the penalties from repeatedly writing to the SD card or wirelessly transmitting. The power can also be reduced with adjusting the duty cycling, which would have the systems at idle in sleep mode for longer periods of time. Both of these methods, however, come at the increased risk of data loss in the case of a system failure (since the cache is stored in the microcontroller’s memory) and a decrease in data resolution.

## 4. Conclusions and Future Work

We introduced a multimodal sensor system for agri- and aquaculture applications. This system provides key features to support scaled-up, distributed, interdisciplinary sensing applications in the field. First, the system design was centered around modular expansion capabilities, both in regards to connectivity and power, to support multiple sensors as well as data storage and transmission methods. This allows it to be used in multiple roles without the need for an extensive redesign and assembly, reducing the cost for development and deployment. The primary limitation in this regard is the enclosure size, but this can be addressed by the printing of a larger design. Second, the improved electronics and enclosure target safe and reliable operation in a variety of conditions, which were tested in simulated and greenhouse environments. Finally, this system utilizes various design techniques to reduce the overall power consumption of the system. This makes additional power available to add more sensors, increase data resolution, and push sampling rates higher up to a continuous draw of 300 mA. An alternate use of this power conservation is to reduce the battery and solar panel sizes in order to decrease deployment costs.

This paper presents the preliminary evaluation of the system with future work including verification of the systems’ accuracy in the field. The system will be further characterized by placing moth systems in fields during the growing season and then manually counting the moths in the traps with the count from the devices. The bivalve systems, equipped with visual and chemical sensors, will be placed by streams and lakes. This sensor fusion will shed more light upon freshwater bivalve movement and behavior. Furthermore, this system’s modularity will be used as the basis for rapid development of new systems targeting new agri- and aqua-cultural and environmental monitoring applications.

## Figures and Tables

**Table 1:** A comparison of commercially available sensor platforms.

Manufacturer	Sensors	Approx. System Cost (USD)
Sencrop	Wind, Temperature, Humidity.	USD 370+
Davis Instruments	Soil Moisture, Temperature.	USD 655
Agrela Ecosystems	Wind, Baro. Pressure, Rainfall, Temp., Humidity, Imaging, Soil Moisture and Temp.	USD 3500 (for base package)
METER	Wind, Baro. Pressure, Rainfall, Temp., Humidity, Canopy Reflectance, Soil Moisture and Temp.	USD 650 + Sensors
Campbell Scientific	Wind, Baro. Pressure, Rainfall, Temp., Humidity, Imaging, Soil Moisture and Temp, Icing, etc.	USD 1340 + Sensors

**Table 2:** System specifications.

Attributes	Values
PCB dimensions	76 mm × 76 mm × 1.6 mm
Cage dimensions	20.7 cm o.d. × 35.5 cm
Supply voltage	3.3 to 12 V
Operating temperature range	0 to 65 ${}^{\circ }C$
Temperature accuracy	0.01 ${}^{\circ }C$
Humidity accuracy	±3% r.H.
Pressure accuracy	0.12 Pa
Temperature measurement range	−40 to 85${}^{\circ }C$
Humidity range	0 to 100% r.H.
Pressure range	300 to 1100 hPa
Moth Detector wavelength	940 nm
Supported wireless protocol	LoRa
Wireless max sensitivity	−148 dBm

**Table 3:** Major components and their cost.

Component	Purpose	Approximate Cost Each per 25 Systems as of 2022
STML073	Microcontroller	$5.90
LTC3106	Solar power regulator	$7.49
TPS22918	Power switch	$1.01
MIC2288	Boost regulator	$0.58
BME280	Gas, humidity, pressure, temperature sensor	$10.65
APT1608F3C	IR emitter	$0.33
TSOP36238TR	IR sensor	$1.47
1733	Anemometer	$44.95
16GB SDHC	Memory	$6.40
3231	LoRa radio module	$17.96
LSM303AGR	Accelerometers	$45.68
PCA9547	Multiplexer	$2.38
503583	Battery	$12.89
925-1418	Enclosure	$18.39
X001452EHN	2.5 W solar panel	$5.94

**Table 4:** Moth system events energy consumption characterization.

Moth System Event	Avg. Current (mA)	Duration per Hour (s)	Energy Consumption (μWh)
Sleep	5.4	3554.1	26,394.4
Moth detection	10.9	2.8	42.1
Sensor timeout	6.1	27.7	233.6
Weather measurement	49.7	15.4	1060.8

**Table 5:** Bivalve system events power consumption characterization.

Bivalve System Event	Avg. Current (mA)	Duration per Hour (s)	Energy Consumption (μWh)
Sleep	3.3	3588.8	16,528.2
LoRa environmental TX	155.9	0.8	180.6
Angle measurement	11.4	8.7	138.5
LoRa angle TX	148.4	1.7	342.5

## Data Availability

Publicly available datasets were analyzed in this study. These data can be found here: https://go.ncsu.edu/mcausuy (accessed on 13 July 2022).

## Abbreviations

The following abbreviations are used in this manuscript:

IoT	Internet of Things
IO	Input/Output
IR	Infrared
IMU	Inertial Measurement Unit
ISM	Industrial, Scientific, and Medical
SNR	Signal-to-Noise Ratio
LC	Resonant Circuit
DC	Direct Current
LoRa	Long-Range Radio
RTC	Real-Time Clock
LED	Light Emitting Diode
ADC	Analog-to-Digital Converter
RH	Relative Humidity
TX	Transmission
PWM	Pulse Width Modulation
PCB	Printed Circuit Board
